# Supplementary material for: Evidence-driven spatiotemporal COVID-19 hospitalization prediction with Ising dynamics
Source: Nat Commun. 2023 May 29;14:3093. doi: 10.1038/s41467-023-38756-3 (PMC10226446; doi:10.1038/s41467-023-38756-3)
Supplement: Supplementary file 1 — Supplementary Information File [file 41467_2023_38756_MOESM1_ESM.pdf]

Supplementary Information

Predicted state-level hospitalization curve by HOIST

To present a general visualization for the predicted curves, we aggregate the county-level predictions into a state-level view. We plot these predicted hospitalization curves for all 50 states and 2 territories (District of Columbia and Puerto Rico) in the US in **Supplementary Figure 1**. All figures are segmented into three distinct time phases to create the training, validation, and testing phases. The curves are the ground truth curve (the blue line) and HOIST's predicted curve (the pink line).

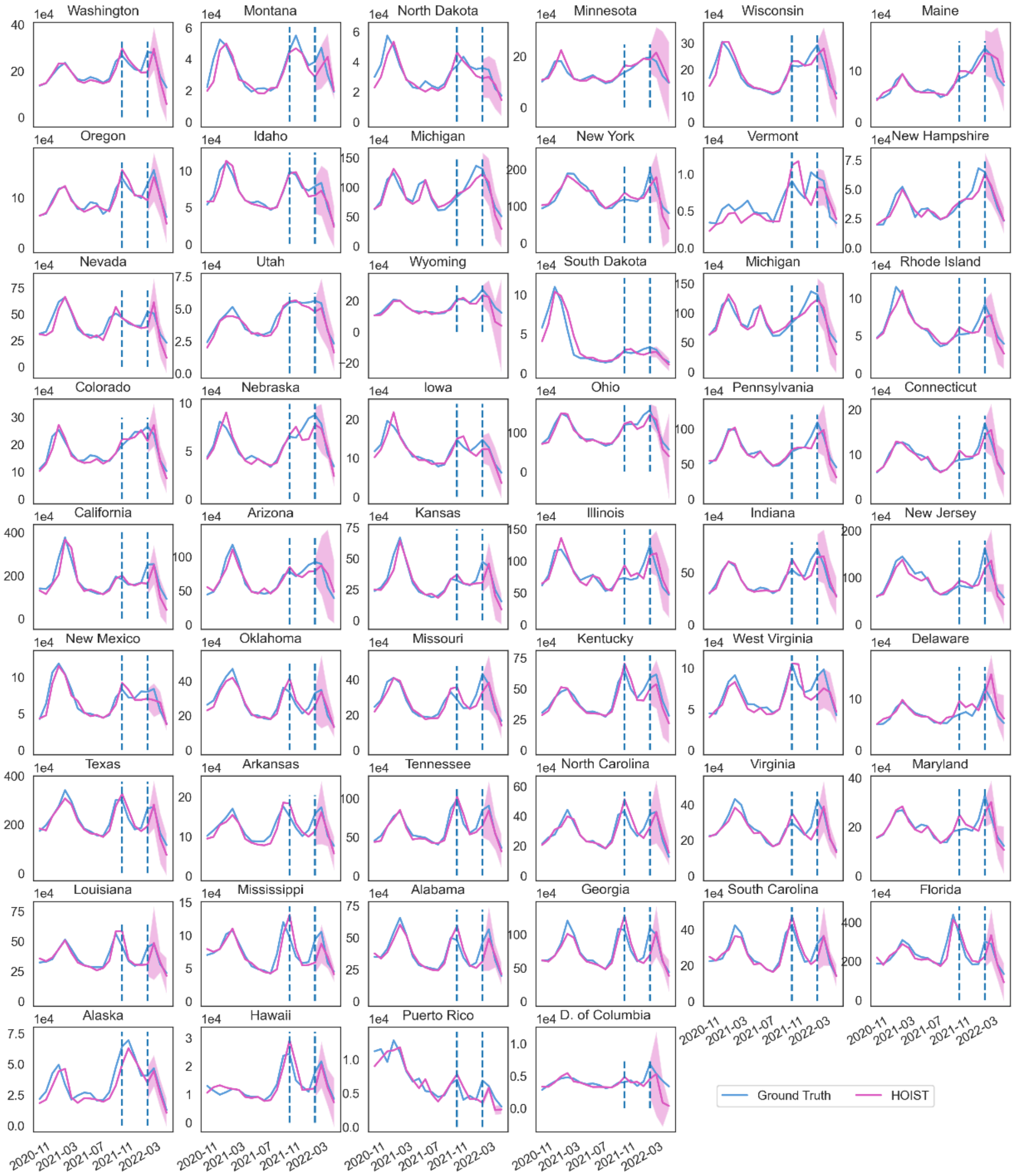

Supplementary Figure 1 Predicted hospitalization curve for all 42 states. The line denotes the mean value and the shadowed area denotes the prediction interval with 90% confidence over 5 random initializations. The state plots are ordered by the relative geographic locations. Source data are provided

as a Source Data file.

These figures show that the predicted curves generally match the ground-truth curves well, suggesting that HOIST accurately predicts not only long-term trends but also short-term variations. The full county-level prediction plots are available on the online visualization platform.

Visualizations of Learned Spatial Embeddings

In order to show the learned spatial embeddings, we use the T-SNE to reduce the learned location embeddings $\mathbf{z}_{i,t}$ to two dimensions. The visualization results are shown in **Supplementary Figure 2**.

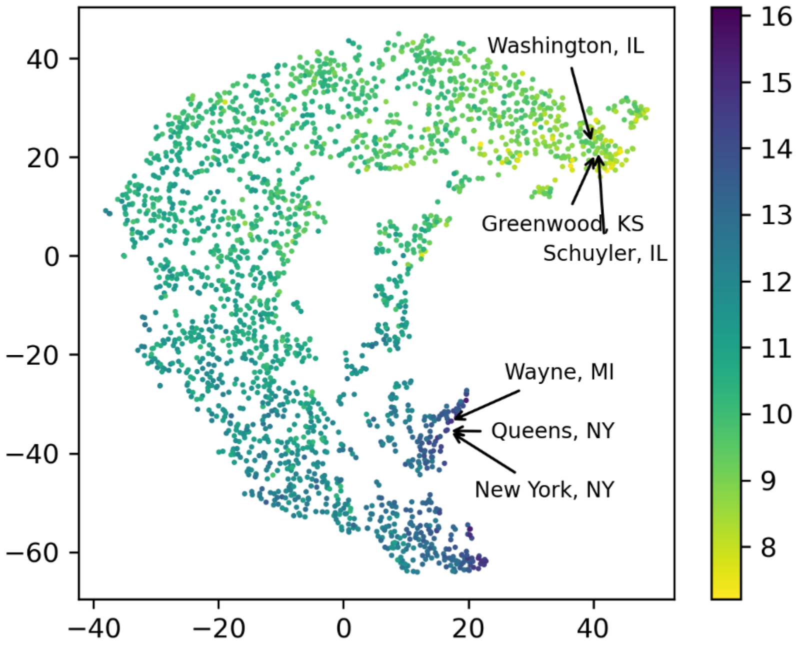

Supplementary Figure 2 T-SNE visualization of learned spatial embeddings. Each dot indicates a location. The color indicates the population size in log scale. We provide six example locations in the figure. Source data are provided as a Source Data file.

The results show that HOIST can learn clustered spatial embeddings using the demographics data. HOIST can capture the similarity between large metropolises and small cities. We also select a few cities and find the nearest cities in the latent space. For New York County, the top 5 nearest counties are Queens NY, Kings NY, Wayne MI, King WA and Palm Beach FL. All five counties are either close to New York or metropolis that have more than 1 million populations. Similarly, for Los Angeles County, the top 5 nearest counties are Kings NY, Queens NY, Philadelphia PA, Maricopa AZ and New York NY. For small counties such as Schuyler IL, which only has a population of 7,544, the nearest locations are Washington IL, Greenwood KS and Sac IA, where Washington is next to Schuyler and the latter two locations both have populations less than 10,000. These results show that HOIST can not only extract geographical similarities between locations, but also can identify geographically distant but socio-economically similar locations. We also made an interactive T-SNE plot online at https://v1xerunt.github.io/HOIST/.

Feature Statistics

The features used in this work and detailed statistics are shown in **Supplementary Table 1**,

Supplementary Table 2, **Supplementary Table 3** and **Supplementary Table 4**.

Supplementary Table 1 Feature lists of population demographics, economics, and healthcare statistics, and medical resource usage statistics.

Feature category	Feature details
Population demographics $\mathbf{M} \in \mathbb{R}^{N\times 9}$	Total population; Populations by race and ethnicity group (Black, White, Asian, Hispanic, Not Hispanic); Populations by age group (0~17, 18~64, >65)
Economics and healthcare statistics $\mathbf{E} \in \mathbb{R}^{N\times 4}$	Number of hospitals, Number of ICU Beds, Average annual income, Unemployment rate

Supplementary Table 2 Overview of location-level statistics for daily hospitalizations, number of visits, and patients with high-risk conditions in tensor $\mathbf{C} \in \mathbb{R}^{N\times T\times 20}$. We first calculate the average value over all timesteps for each feature in each location. We then report the mean, median, and max values of all locations. Source data are provided as a Source Data file.

Feature name	Mean	Median	Max
Hospitalization	31	2	2,878
Number of visits	315	46	24,776
Number of visits, age >= 65	159	24	10,924
Cerebrovascular disease	4	1	271
Chronic lung disease	14	3	746
Congestive heart failure	17	3	1,096
Dementia	13	2	517
Diabetes without chronic complication	24	4	2,315
HIV	3	0	269
Hemiplegia or paraplegia	2	0	106
Hypertension	27	4	1,761
Immunodeficiency	1	0	90
Liver disease	2	0	254
Malignancy	6	0	456
Metastatic solid tumor	1	0	104
Myocardial infarction	1	0	106
Obesity	5	1	403
Peptic ulcer disease	0	0	25
Peripheral vascular disease	5	1	353
Renal disease	21	2	1,694

Supplementary Table 3 Overview of location-level statistics for daily vaccination shots in tensor $\boldsymbol{V} \in \mathbb{R}^{N\times T\times 17}$. We first calculate the average value over all timesteps for each feature in each location. We then report the mean, median, and max values of all locations. Source data are provided as a Source Data file.

Feature name	Mean	Median	Max
Total shots	22	2	5,497
Total first shots	8	0	2,583

Total second plus shots	7	0	2,345
Total booster shots	8	1	1,553
Pfizer 1st shots	4	0	1,254
Pfizer 2nd shots	3	0	1,210
Pfizer booster shots	4	0	778
Moderna 1st shots	3	0	1,060
Moderna 2nd shots	2	0	1,104
Moderna booster shots	3	0	667
Johnson & Johnson's Janssen 1st shots	1	0	227
Johnson & Johnson's Janssen booster shots	0	0	18
Pfizer tris-sucrose 30mcg/0.3mL 1st shots	0	0	9
Pfizer tris-sucrose 30mcg/0.3mL 2nd shots	0	0	10
Pfizer tris-sucrose 30mcg/0.3mL booster shots	1	0	106
Pfizer tris-sucrose 10mcg/0.2mL 1st shots	1	0	49
Pfizer tris-sucrose 10mcg/0.2mL 2nd shots	1	0	44

Supplementary Table 4 Overview of location-level statistics for daily vaccinations in tensor $\mathbf{U} \in \mathbb{R}^{N \times T \times 4}$. We first calculate the average value over all timesteps for each feature in each location. We then report all locations' mean, median, and max values. Source data are provided as a Source Data file.

Feature name	Mean	Median	Max
Usage of hospital bed	217	25	13,262
Usage of hospital bed for COVID-19 patients	26	4	1,746
Usage of ICU bed	33	4	2,126
Usage of ICU bed for COVID-19 patients	6	1	377

Error Analysis

We also conduct the error analysis for MAE and R^2 by showing two counties where HOIST has low R^2 and high MAE in **Supplementary Figure 3**.

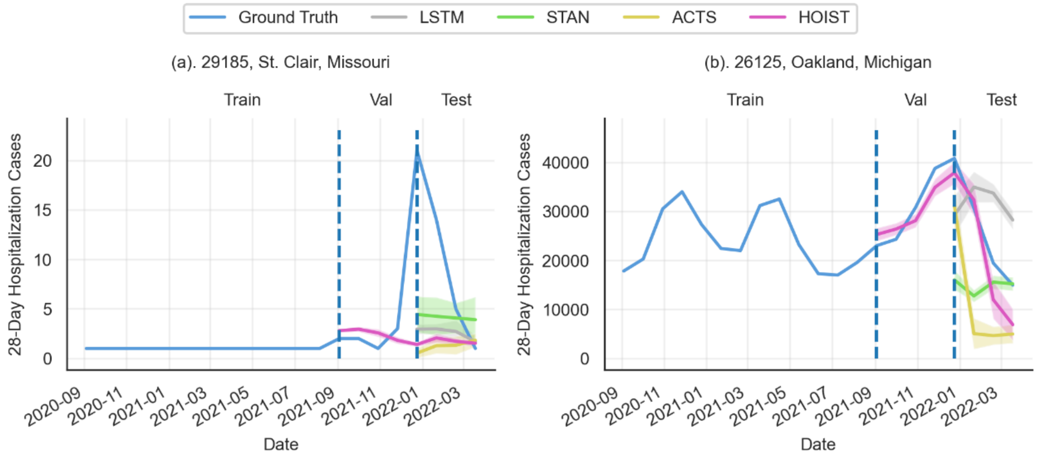

Supplementary Figure 3 Predicted hospitalization curves of two counties. The HOIST achieves low R^2 (left) and high mean average error (MAE) (right) on these two counties. The line denotes the mean value and the shadowed area denotes the prediction interval with 90% confidence over 5 random

initializations. Source data are provided as a Source Data file.

We find that HOIST often achieves a lower R^2 score in locations with low case counts and high case count variability (e.g., a sudden peak in Jan 2022). For the 100 locations in which HOIST achieves the lowest R^2 score, the average 28-day hospitalization cases were 5. HOIST and all baseline models failed to predict this peak. This is possibly an outlier event related to the bias in the claims data, which we further discuss as a limitation in the section below. HOIST achieves its highest MAE in locations with very high case counts, and HOIST can accurately predict the curve's trends with high R^2 scores. For the 100 locations in which HOIST achieves the highest MAE, the average 28-day hospitalization cases were 12,581 and the average R^2 score was 0.63. However, since the case counts are very high, some minor deviations along the curve can lead to high prediction error, though all baseline models perform much worse than HOIST. Though the absolute prediction errors for these locations are high, the accuracy of the trend prediction can still prove useful in providing advanced notice for anticipated surges in hospitalization rates.

Prediction Uncertainty Analysis

To estimate the prediction uncertainty of the models, we use the conformal estimation algorithm. The results show that when the model has 95% confidence on the validation set, the test set coverage rate is 89.3%, which is highly consistent. We also compare the interval width (i.e., $s\sigma$) of 5 baseline models with the highest prediction performance at 90% coverage rate on the test set. The results are shown in **Supplementary Figure 4**.

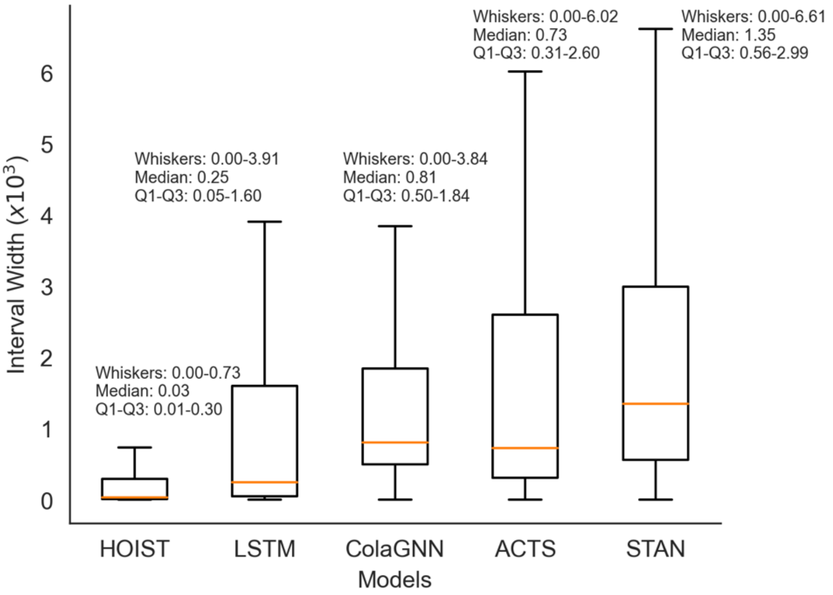

Supplementary Figure 4 Model prediction interval width when the models achieve 90% coverage rate on the test set. The proposed HOIST model achieves a much smaller width compared to other baselines. The box plots are derived from n=2,299 locations over 5 random initializations. Source data are provided as a Source Data file.

The results show that HOIST achieves a much narrower prediction interval compared to other baselines, which indicates that HOIST can achieve precise and accurate prediction with high confidence. This may help make correct and timely decisions in the real world.
